# Therapeutic compliance of bipolar women during the perinatal period

**DOI:** 10.1192/j.eurpsy.2023.1452

**Published:** 2023-07-19

**Authors:** B. Andrea

**Affiliations:** university psychiatry of marseilles, Sainte Margeurite, Marseille, France

## Abstract

**Introduction:**

Bipolar disorder (BD) is a chronic and disabling disease. Its prognosis is largely conditioned by tretment adherence. The pregnancy and post partum are considered as a period of high vulnerability and risk of relapse. According to the literature, no study has investigated treatment adherence in this population.

**Objectives:**

The purpose of this study was to compare medication adherence among 3 groups of bipolar patients: in the postpartum period, one year before the conception , and outside the perinatal period. As a secondary objective, we compared intentional and unintentional adherence among these three groups of patients, as well as adherence dimmensions related to behaviors, attitudes and tolerance.

We also studied concerns and perceived need for treatment and the presence of potential confounders.

**Methods:**

This is a post hoc study conducted using a research protocol entitled RsBip, (NCT03595670), descriptive, mono-centric and open. Patients with a diagnosis of bipolar disorder established by a psychiatrist, having a pregnancy project, pregnant, or having given birth since less than one year, and their age-matched controls were included in analysis. All the patients are recruited within the expert center for bipolar disorders in the University Hospital of Marseille. Standardized and validated questionnaires such as the MARS, the BMQ and the HADS were used.

**Results:**

112 patients participated in the RsBip study. After exclusion of men, women over 50 years of age and women who did not answer the gyneco-obstetrical history questionnaire, 46 patients with a diagnosis of bipolar disorder were included in our study. Among them, 12 patients had a pregnancy plan within the year, 3 patients were pregnant at the time of inclusion, 8 patients had given birth within the year, and 23 patients constituted the “control” group. The characteristics of the population were similar.

Our main hypothesis was partly confirmed since compliance with treatment decreases in the postpartum period, and more precisely intentional compliance and dimensions related to behaviors and attitudes.

**Conclusions:**

Post-partum period is associated with low adherence in BD. The implementation of a specific therapeutic education for patients and the promotion of a shared medical decision as soon as the pregnancy is planned could be proposed.

**Disclosure of Interest:**

None Declared

